# Radiomics Models for the Preoperative Prediction of Pelvic and Sacral Tumor Types: A Single-Center Retrospective Study of 795 Cases

**DOI:** 10.3389/fonc.2021.709659

**Published:** 2021-09-09

**Authors:** Ping Yin, Xin Zhi, Chao Sun, Sicong Wang, Xia Liu, Lei Chen, Nan Hong

**Affiliations:** ^1^Department of Radiology, Peking University People’s Hospital, Beijing, China; ^2^Department of Pharmaceuticals Diagnosis, GE Healthcare, Shanghai, China

**Keywords:** radiomics, machine learning, neoplasms, classification, diagnostic imaging

## Abstract

**Purpose:**

To assess the performance of random forest (RF)-based radiomics approaches based on 3D computed tomography (CT) and clinical features to predict the types of pelvic and sacral tumors.

**Materials and Methods:**

A total of 795 patients with pathologically confirmed pelvic and sacral tumors were analyzed, including metastatic tumors (n = 181), chordomas (n = 85), giant cell tumors (n =120), chondrosarcoma (n = 127), osteosarcoma (n = 106), neurogenic tumors (n = 95), and Ewing’s sarcoma (n = 81). After semi-automatic segmentation, 1316 hand-crafted radiomics features of each patient were extracted. Four radiomics models (RMs) and four clinical-RMs were built to identify these seven types of tumors. The area under the receiver operating characteristic curve (AUC) and accuracy (ACC) were used to evaluate different models.

**Results:**

In total, 795 patients (432 males, 363 females; mean age of 42.1 ± 17.8 years) were consisted of 215 benign tumors and 580 malignant tumors. The sex, age, history of malignancy and tumor location had significant differences between benign and malignant tumors (*P* < 0.05). For the two-class models, clinical-RM2 (AUC = 0.928, ACC = 0.877) performed better than clinical-RM1 (AUC = 0.899, ACC = 0.854). For the three-class models, the proposed clinical-RM3 achieved AUCs between 0.923 (for chordoma) and 0.964 (for sarcoma), while the AUCs of the clinical-RM4 ranged from 0.799 (for osteosarcoma) to 0.869 (for chondrosarcoma) in the validation set.

**Conclusions:**

The RF-based clinical-radiomics models provided high discriminatory performance in predicting pelvic and sacral tumor types, which could be used for clinical decision-making.

## Introduction

Pelvic and sacral tumors have various types, with metastatic tumors being the most common because of their prominent hematopoietic function until late in life (1, 2). Primary sacral tumors are rare and mainly include chordoma, giant cell tumor (GCT), neurogenic tumor, etc. (3, 4). In addition, a small number of GCTs and neurogenic tumors can also occur in the pelvis. Sarcomas, such as chondrosarcoma, osteosarcoma, and Ewing’s sarcoma, are also the common primary malignant bone tumors of the pelvis and sacrum (5, 6). Because pelvic and sacral tumors are usually large and surrounded by complex structures, treatment for these tumors is often a challenging procedure that can be accompanied by serious complications, such as massive bleeding (7–10). In clinical practice, these tumors are treated differently. For example, preoperative chemotherapy is important for osteosarcoma, but it is not effective for chondrosarcoma. Therefore, accurate preoperative identification of these tumors is essential for the development of individualized treatment (11, 12).

Pelvic and sacral tumors share many similar clinical and imaging features, they often present heterogeneous masses with different components. This makes it difficult to identify these tumors in clinical practice, especially when they occur in unusual or multiple locations. In addition, the classic semantic assessments of traditional computed tomography (CT) and magnetic resonance (MR) images suffer from strong inter- and intra-reader variations, they do not provide sufficient diagnostic power to identify these tumors (13). Clinically, a simple and accurate method is needed to identify pelvic and sacral tumors.

Sophisticated machine learning methods show promise in complementing human diagnostics (14, 15). In recent years, radiomics has been successfully applied in the classification of tumor types. A few previous studies have identified sacral tumors using radiomics methods, but most of these studies identified only two types of tumors, and their sample sizes were relatively small (3, 11, 12). Yin et al. (3) built a random forest (RF)-based triple-classification radiomics model for the differentiation of primary chordomas, GCTs, and metastatic tumors of sacrum based on MR features. They concluded that their model is feasible to differentiate these tumors and can improve the precision of preoperative diagnosis in clinical practice. RF algorithms have a comparably low tendency to overfit and are well suited for multi-classification discrimination (13). Although machine learning has been applied to the segmentation, lesion detection, evaluation of chemotherapy response and prediction of local recurrence of bone tumors in recent years (16–20), the ability to identify multiple types of pelvic and sacral tumors remains unknown.

The aim of our study was to determine the performance of RF-based radiomics approaches based on CT features and clinical characteristics to predict the multiple types of pelvic and sacral tumors.

## Materials and Methods

### Patients and Data Acquisition

After the approval of our local ethics committee, we conducted this single-center retrospective study and waived written informed consent. A total of 1000 patients with pathologically confirmed pelvic and sacral tumors in our institution from April 2006 to December 2019 were retrospectively analyzed. All patients had a single pelvic and sacral tumor that was detected on CT within 1 month before the initial surgery. Patients that had pelvic and sacral tumor types with a sample size less than 30 (n = 111), without preoperative CT images (n = 84), or with obvious artifacts (n = 10) were excluded. Finally, a total of 795 patients with pelvic and sacral tumor types of metastatic tumors (n = 181), chordomas (n = 85), GCTs (n =120), chondrosarcomas (n = 127), osteosarcomas (n = 106), neurogenic tumors (n = 95), or Ewing’s sarcomas (n = 81) were included in the study. Sex, age, maximal tumor size, history of malignancy and tumor location (Zone I–IV) (21) of patients were also analyzed. Zone I includes the iliac crest, Zone II includes the acetabulum and its surroundings, Zone III includes the pubis and ischium regions, and Zone IV refers to the sacrum region. A “multi-zone” is a tumor that involves more than one area simultaneously.

All CT images were acquired on each patient using multi-detector row CT systems (Philips iCT 256, Philips Medical System; GE Lightspeed VCT 64, GE Medical System). The acquisition parameters were as follows: 120 kV, 100-370 mAs, slice thickness = 5 mm, matrix = 512 × 512 mm, field of view = 350 × 350 mm. The CT images were reconstructed with soft-tissue and bone kernel.

### Tumor Segmentation

MITK software version 2018.04.2 (www.mitk.org) was used for the semi-automatic segmentation of all tumors (22). First, the two authors (PY and XL) manually delineated the edge of the lesion at the axial, sagittal, and coronal sites, respectively. Then, a three-dimensional lesion was automatically formed and manually corrected by a musculoskeletal radiologist with 5 years of experience and a senior musculoskeletal radiologist with 20 years of experience who were blinded to the assessment.

### Feature Extraction and Selection

A total of 1316 radiomics features of each patient were extracted from the CT images using the Artificial Intelligence Kit software version 3.3.0 (GE Healthcare, China), including 18 first-order histogram features, 14 shape features, 24 gray-level co-occurrence matrix features, 14 gray-level dependence matrix features, 16 gray-level run-length matrix features, 16 gray-level size-zone matrix features, 5 neighboring gray-tone difference matrix features, 744 wavelet features, 186 Laplacian of Gaussian (LoG_sigma=2.0/3.0_) features, and 279 local binary pattern features.

We preprocessed the data and normalized the extracted features. When the data value exceeded the range of mean value and standard deviation, the median of specific variance vector was used to replace the outliers. In addition, we standardized the data in a specific interval. The standardized formula is as follows: (fi−u)/std, where fi represents a single characteristic data, u is the average value of the data column, and std pertains to the standard deviation of the data column.

To reduce overfitting or selection bias in our radiomics model, we used two methods to select the features: Spearman correlation as representative of filter models and gradient boosting decision tree (GBDT) as representative of embedded models (23, 24). The features with Spearman correlation > 0.7 were excluded to avoid overfitting. After the number of features was determined, the most predictive radiomics features were chosen to construct the final model.

### Model Building and Validation

First, we divided the patients into the benign tumors group (n=215) and malignant tumors group (n=580), and built the first radiomics model (RM1) by using RF. After differentiating benign and malignant tumors, the specific types of benign and malignant tumors were then divided respectively. RM2, a two-class RF-based radiomics model, was built to identify GCTs and neurogenic tumors. After that, we constructed two triple-classification models, namely, RM3 and RM4. RM3 was used to identify metastatic tumors, chordomas, and sarcomas. RM4 was used to identify osteosarcomas, chondrosarcomas, and Ewing’s sarcomas.

Clinical features were also compared and variables with *P* value < 0.05 were included in the clinical model. When combined RM with clinical data, we also constructed four clinical-RMs. In addition, we also constructed a seven-classification model, namely, clinica-RM5, to identify these seven types of tumors. In each model, all patients were randomly divided the patients into the training and validation sets by a ratio of 7:3. Models were trained with the training set by using the repeated 5-fold cross-validation method, and estimation performance was evaluated with the validation set.

**Figure 1:**
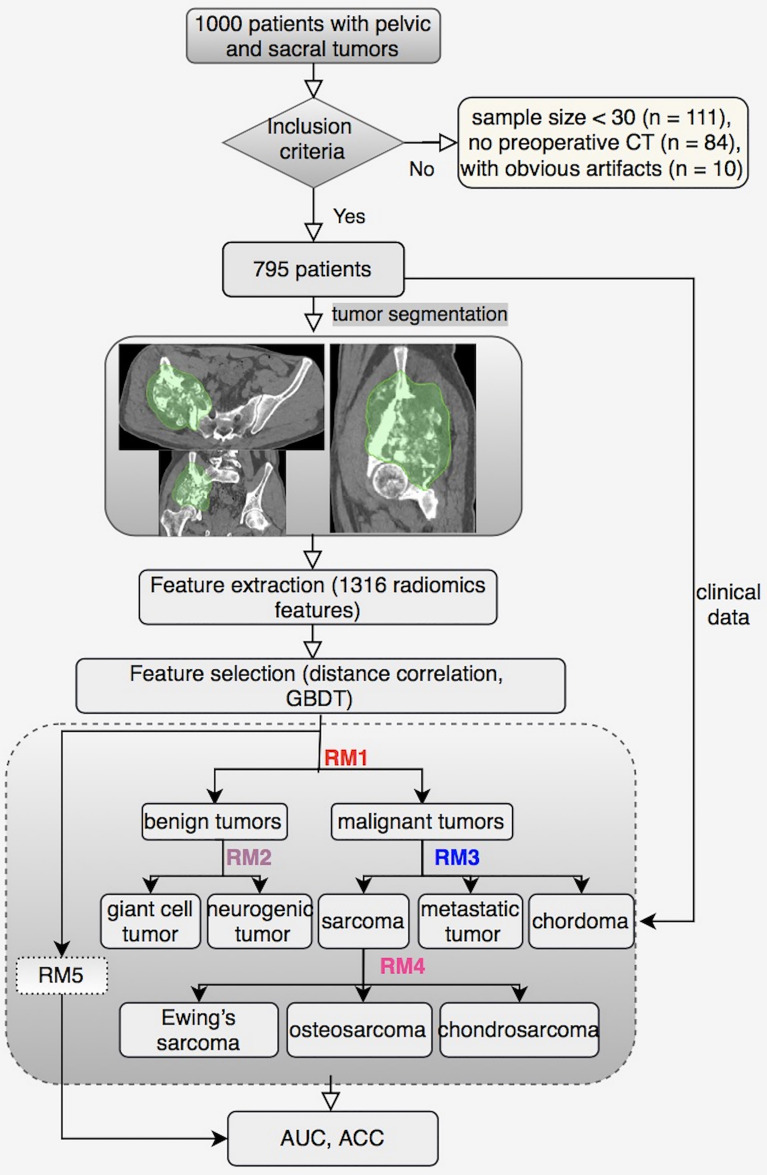
The workflow of this study.

The performance of different models was assessed using the area under the receiver operating characteristic curve (AUC) and accuracy (ACC). **Figure 1** shows the workflow of this study.

### Statistical Analysis

All statistical analyses were performed with R (version 3.5.1) and Python (version 3.5.6). Mann-Whitney U test or Kruskal-Wallis H test was performed to compare continuous variables, while chi-squared test or Fisher’s exact was used for categorical variables between groups. All statistical tests were two-sided, and Bonferroni-corrected *P* value were used to identify the feature significance of multiple comparisons.

## Results

### Patient Characteristics

A total of 795 patients (432 males, 363 females; mean age of 42.1 ± 17.8 years, range 4–82 years; mean tumor size of 9.6 ± 4.0 cm) were included in this study (**Table 1**). In the clinical-RM1, sex, age, history of malignancy and tumor location had significant differences between groups (*χ^2^* = 9.111, *Z* = -3.962, *χ^2^* = 62.277, *χ^2^* = 149.379, *P* < 0.01). No significant difference was found in terms of tumor size between groups (*Z* = 0.534, *P* > 0.05). For the clinical-RM3, age, tumor size, history of malignancy and tumor location had significant differences between groups (*Z* = 248.6, *Z* = 55.167, *χ^2^* = 272.494, *χ^2^* = 181.17, *P* < 0.001). However, for the clinical-RM2 and clinical-RM4, significant differences were found in terms of age, tumor size and tumor location (*P* < 0.01), no significant difference was found in terms of sex and history of malignancy between groups (*P* > 0.05). There was no significant statistical difference between the training and validation sets in terms of age, sex, tumor location, tumor size, history of malignancy and histology (*P* > 0.05). The 95 neurogenic tumors were composed of 45 neurofibromas and 50 schwannomas. In the case of metastatic tumor, 43 metastases from lung, 27 from breast, 26 from liver, 25 from kidney, 15 from gastrointestinal, 13 from thyroid, 12 from prostate, 9 from uterus, 4 from bladder, 2 from melanoma, 2 from osteosarcoma, 2 from brain, and 1 from nasopharynx, respectively.

**Table 1:** Clinical characteristic of patients.

Variable	Class 1	Class 2	Class 3	Statistics	*P* value
Clinical-RM1					
Age (years)	36.00 (27.20, 48.00)	47.00 (27.00, 59.00)	–	-3.962	<0.001
Size (cm)	8.90 (7.02, 11.80)	9.00 (6.50, 12.10)	–	0.534	0.593
Female	117 (54.42%)	246 (42.41%)	–	9.111	0.003
Male	98 (45.58%)	334 (57.59%)	–		
Location I	10 (4.65%)	123 (21.21%)	–	149.379	<0.001
Location II	3 (1.40%)	49 (8.45%)	–		
Location III	3 (1.40%)	47 (8.10%)	–		
Location IV	186 (86.51%)	219 (37.76%)	–		
Multi-location	13 (6.05%)	142 (24.48%)	–		
No history of malignancy	211 (98.14%)	422 (72.76%)		62.277	<0.001
A history of malignancy	4 (1.86%)	158 (27.24%)			
Clinical-RM2					
Age (years)	44.00 (32.00, 53.80)	33.00 (25.00, 43.00)	–	4.726	<0.001
Size (cm)	8.50 (6.40, 11.66)	9.20 (7.80, 12.10)	–	-2.129	0.033
Female	51 (53.68%)	66 (55.00%)	–	0.037	0.847
Male	44 (46.32%)	54 (45.00%)	–		
Location I	1 (1.05%)	9 (7.50%)	–	–	0.005
Location II	0 (0.00%)	3 (2.50%)	–		
Location III	0 (0.00%)	3 (2.50%)	–		
Location IV	91 (95.79%)	95 (79.17%)	–		
Multi-location	3 (3.16%)	10 (8.33%)	–		
No history of malignancy	93 (97.89%)	118 (98.33%)		0.074	0.786
A history of malignancy	2 (2.11%)	2 (1.67%)			
Clinical-RM3					
Age (years)	29.00 (19.00, 45.05)	58.00 (50.00, 65.00)	59.00 (49.70, 68.30)	248.6	<0.001
Size (cm)	10.20 (7.39, 13.11)	7.60 (5.50, 9.80)	8.10 (6.20, 11.06)	55.167	<0.001
Female	133 (42.36%)	85 (46.96%)	28 (32.94%)	4.656	0.098
Male	181 (57.64%)	96 (53.04%)	57 (67.06%)		
Location I	81 (25.80%)	42 (23.20%)	0 (0.00%)	181.17	<0.001
Location II	29 (9.24%)	20 (11.05%)	0 (0.00%)		
Location III	35 (11.15%)	12 (6.63%)	0 (0.00%)		
Location IV	64 (20.38%)	71 (39.23%)	84 (98.82%)		
Multi-location	105 (33.44%)	36 (19.89%)	1 (1.18%)		
No history of malignancy	298 (94.90%)	50 (27.62%)	74 (87.06%)	272.494	<0.001
A history of malignancy	16 (5.10%)	131 (72.38%)	11 (12.94%)		
Clinical-RM4					
Age (years)	17.00 (13.00, 25.30)	26.00 (19.00, 34.05)	44.00 (33.00, 52.00)	127.47	<0.001
Size (cm)	8.00 (5.41, 10.26)	11.75 (8.00, 14.41)	11.00 (8.62, 13.58)	31.792	<0.001
Female	26 (32.10%)	47 (44.34%)	60 (47.24%)	4.904	0.086
Male	55 (67.90%)	59 (55.66%)	67 (52.76%)		
Zone I	23 (28.40%)	28 (26.42%)	30 (23.62%)	24.453	0.002
Zone II	5 (6.17%)	8 (7.55%)	16 (12.60%)		
Zone III	10 (12.35%)	8 (7.55%)	17 (13.39%)		
Zone IV	28 (34.57%)	16 (15.09%)	20 (15.75%)		
Multi-zone	15 (18.52%)	46 (43.40%)	44 (34.65%)		
No history of malignancy	76 (93.83%)	99 (93.40%)	123 (96.85%)	1.687	0.43
A history of malignancy	5 (6.17%)	7 (6.60%)	4 (3.15%)		

Clinical-RM1: class 1 = benign tumor, class 2 = malignant tumor; Clinical-RM2: class 1 = neurogenic tumor, class 2 = giant cell tumor; Clinical-RM3: class 1 = sarcoma, class 2 = metastatic tumor, class 3 = chordoma; Clinical-RM4: class 1 = Ewing’s sarcoma, class 2 = osteosarcoma, class 3 = chondrosarcoma.

### Performance of Different Models

**Figure 2:**
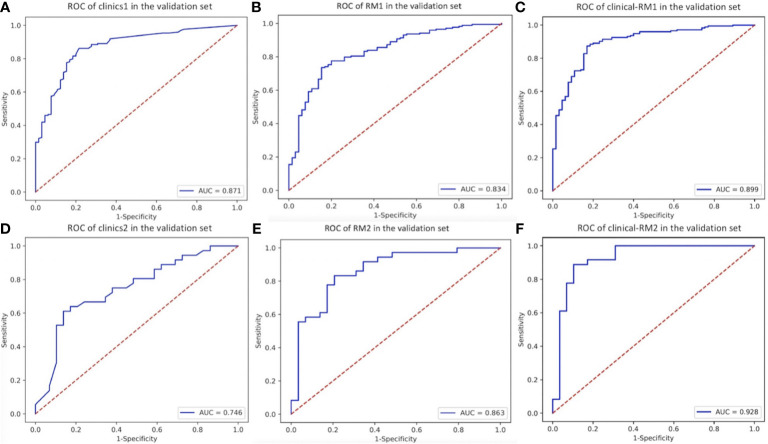
The ROC curve of two-class models in the validation set. **(A)**, clinics1. **(B)**, RM1. **(C)**, clinical-RM1. **(D)**, clinics2. **(E)**, RM2. **(F)**, clinical-RM2.

For the two-class models, RM1 achieved an AUC of 0.949 and an ACC of 0.894, while RM2 reached an AUC of 0.974 and an ACC of 0.913 in the training set (**Figure 2** and **Table 2**). In the validating set, we found: RM2 (AUC = 0.863, ACC = 0.800) had a slightly higher performance than RM1 (AUC = 0.834, ACC = 0.782).

**Table 2:** Performance of two-class models in the validation set.

	AUC	ACC	Sensitivity	Specificity	PPV	NPV
RM1	0.834	0.782	0.891	0.492	0.824	0.627
RM2	0.863	0.800	0.917	0.655	0.767	0.864
Clinics1	0.871	0.833	0.874	0.723	0.894	0.681
Clinics2	0.746	0.646	0.833	0.414	0.638	0.667
Clinical-RM1	0.899	0.854	0.948	0.600	0.864	0.812
Clinical-RM2	0.928	0.877	0.889	0.862	0.889	0.862

AUC, area under curve; ACC, accuracy; PPV, positive predictive value; NPV, negative predictive value.

**Figure 3:**
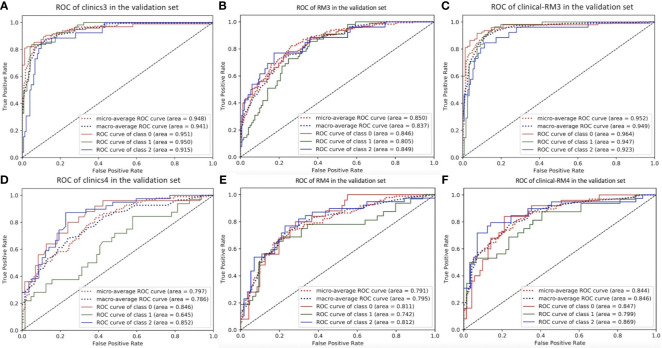
The ROC curve of three-class models in the validation set. **(A)**, clinics3. **(B)**, RM3. **(C)**, clinical-RM3. **(D)**, clinics4. **(E)**, RM4. **(F)**, clinical-RM4. For clinics3, RM3 and clinical-RM3: class 0 = sarcoma, class 1 = metastatic tumor, class 2 = chordoma. For clinics4, RM4 and clinical-RM4: class 0 = Ewing’s sarcoma, class 1 = osteosarcoma, class 2 = chondrosarcoma.

For the three-class models, the proposed radiomics classifiers achieved AUCs between 0.742 (for osteosarcoma, RM4) and 0.849 (for chordoma and sarcoma, RM3) in the validation set (**Figure 3** and **Table 3**). The ACCs of RM3 and RM4 were 0.665 and 0.667 in the validation set, respectively.

**Table 3:** Performance of three-class models in the validation set.

	AUC	ACC	Precision	Recall	F1-score
RM3					
metastatic tumor	0.805	0.665	0.645	0.364	0.465
chordoma	0.849	0.665	0.818	0.346	0.486
sarcoma	0.846	0.665	0.657	0.926	0.769
RM4					
chondrosarcoma	0.812	0.667	0.682	0.769	0.723
osteosarcoma	0.742	0.667	0.636	0.656	0.646
Ewing’s sarcoma	0.811	0.667	0.684	0.520	0.591
Clinics3					
metastatic tumor	0.950	0.824	0.836	0.836	0.836
chordoma	0.915	0.824	0.609	0.538	0.571
sarcoma	0.951	0.824	0.867	0.895	0.881
Clinics4					
chondrosarcoma	0.852	0.583	0.743	0.667	0.703
osteosarcoma	0.645	0.583	0.448	0.406	0.426
Ewing’s sarcoma	0.846	0.583	0.531	0.680	0.596
Clinical-RM3					
metastatic tumor	0.947	0.841	0.780	0.836	0.807
chordoma	0.923	0.841	0.737	0.538	0.622
sarcoma	0.964	0.841	0.898	0.926	0.912
Clinical-RM4					
chondrosarcoma	0.869	0.667	0.756	0.795	0.775
osteosarcoma	0.799	0.667	0.625	0.625	0.625
Ewing’s sarcoma	0.847	0.667	0.565	0.520	0.542

AUC, area under curve; ACC, accuracy.

The AUC of the clinical models ranged from 0.645 to 0.951, and the ACC ranged from 0.583 to 0.833 in the validation set. When combined with clinical features, clinical-RMs performed better than individual RMs and clinical models. The clinical-RM1 exhibited an AUC of 0.899 and an ACC of 0.854 in the validation set. Similarly, the clinical-RM2 (AUC = 0.928, ACC = 0.877) performed better than individual RM2 in the validation set. The clinical-RM3 achieved AUCs between 0.923 (for chordoma) and 0.964 (for sarcoma), and ACC of 0.841 in the validation set. The AUCs of the clinical-RM4 ranged from 0.799 (for osteosarcoma) to 0.869 (for chondrosarcoma) in the validation set.

In addition, the AUC and ACC of clinical-RM5 in the training set were 0.771 and 0.580, and those in the validation set were 0.722 and 0.533, respectively. The Bar chart reflecting the predictive values of RM5 was shown in **Supplemental Figure 1**.

## Discussion

In this study, we found significant differences in terms of age, sex, history of malignancy and tumor location for differentiating benign and malignant pelvic and sacral tumors. Both two-class and three-class RMs had good performance in predicting pelvic and sacral tumor types. When combined with clinical data, the clinical-RMs performed better than individual RMs.

Pelvic and sacral tumors have a tendency to be silent till reaching extreme volumes and involving the adjacent nerve roots, blood vessels and organs (7). They often present as large heterogeneous masses, which are often difficult to identify on conventional imaging. Although metastatic tumors occur frequently in the pelvis and sacrum, a diagnostic dilemma can present when there is a single tumor with no history or evidence of malignancy elsewhere in the body (1). In our study, age, sex, history of malignancy and tumor location were considered to be important clinical features to differentiate benign and malignant pelvic and sacral tumors. The proportion of males in patients with malignant tumors was higher than that in patients with benign tumors, which is consistent with previous study (12). For the clinical-RM3, age, tumor size, history of malignancy and tumor location had significant differences between groups. For the clinical-RM2 and clinical-RM4, however, significant differences were found in terms of age, tumor size and tumor location. Chordomas of the pelvis and sacrum occur almost exclusively in the sacrum, but can also involve the pelvis when the tumor is large, increasing the difficulty of identification. Neurogenic tumors and GCTs of bone also occur more frequently in the sacrum than in the pelvis. In this study, 4 neurogenic tumors and 25 GCTs occurred in the pelvis. Nevertheless, more sarcomas occur in the pelvis than in the sacrum. Chondrosarcoma is the most common primary malignant bone tumor in the pelvis, followed by osteosarcoma and Ewing’s sarcoma (5, 6). Osteosarcomas and Ewing’s sarcomas tend to occur between the ages of 10 and 30, while chondrosarcoma mostly affects people in their 40s to 70s (6). In our study, we found that the mean age of Ewing’s sarcoma was the lowest among these tumors, which is consistent with previous study (25). Park et al. (2) reported that osteosarcoma of the pelvic bones was more frequent in older patients, which may be due to their limited sample size. GCTs of bone typically affect younger patients between 20 and 30 years, and neurogenic tumors tend to occur between 20 and 50 years old without significant age predominance (4). In line with previous studies (1, 26, 27), we found that metastatic tumors and chordomas were more common in older populations. Furthermore, the size of the GCTs was significantly larger than that of the neurogenic tumor. The size of the sarcoma was significantly larger than that of the metastatic tumor and chordoma. For sarcomas, the size of the Ewing’s sarcoma was significantly smaller than that of the osteosarcoma and chondrosarcoma. As expected, the proportion of patients with metastatic tumors who had a history of malignancy was significantly higher than that of other tumor types.

Until now, there are few studies on the differentiation of pelvic and sacral tumors using machine learning methods (3, 11, 12). Yin et al. (12) compared the performance of radiomics model based on CT and MR features to identify sacral tumors. They found that clinical-radiomics nomogram performed better than radiomics nomogram. Most of previous studies investigated two-class problems, which intrinsically achieve higher AUCs compared with multiclass problems (13). In this study, we built four radiomics classifiers to identify seven types of pelvic and sacral tumors using RF. RF was widely used in multi-class machine learning due to its high accuracy and low overfitting (28, 29). It is a relatively efficient model-free method both in variable selection and classification (3). Kniep et al. (13) built a RF-based five-class radiomics model to predict the metastatic tumor type of brain and found it is superior to the radiologist’s readings. In our study, we first proposed a four-step models framework to improve the identification efficiency of models. Using this multi-model framework, we identified seven types of pelvic and sacral tumors, beginning with benign and malignant tumors and their subgroups. We found that both two-class and three-class RF-based RMs had good performance in predicting pelvic and sacral tumor types. Although the RMs performed worse than the clinical models alone, except for the RM2. This may be related to the fact that the clinical features included in this study are important features for differentiating these tumors and that they differ significantly. When combined with clinical data, the clinical-RMs performed better than individual RMs, which is consistent with previous studies (6, 12). Furthermore, the AUC of the seven-classification model was lower than that of other models, which may be related to the performance of RF decreases as the number of categories increases.

Our study has certain limitations. First, all images were collected from one center over the past decade or so, and from two different machines. Although we included large sample data for the study, a multicenter prospective study is beneficial to future research. Second, only plain CT image data were available in this study. Multimodal data may be required, such as enhanced CT and MR, which may provide more useful information for the differentiation of lesions. Third, we did not compare the performance of our model with that of the radiologists, and we will conduct further research in the following study. Last, we cannot include all types of pelvic and sacral tumors because some types are extremely rare. Our models include seven types of the most common pelvic and sacral tumors, and we continue to believe that our models can be of great clinical benefit.

In conclusion, the RF-based clinical-RMs provided high discriminatory performance in predicting pelvic and sacral tumor types. Our models can provide a simple, non-invasive and accurate auxiliary diagnostic tool for the differentiation of pelvic and sacral tumors, improving the diagnostic efficiency of clinicians.

## Data Availability Statement

The original contributions presented in the study are included in the article/**Supplementary Material**. Further inquiries can be directed to the corresponding author.

## Ethics Statement

The studies involving human participants were reviewed and approved by Peking University People’s Hospital Ethics Committee. Written informed consent was waived.

## Author Contributions

Guarantor of integrity of the entire study: NH. Study concepts: PY and NH. Study design: PY and NH. Definition of intellectual content: PY and XZ. Literature research: PY and XZ. Clinical studies: PY and XZ. Experimental studies: PY and XL. Data acquisition: PY and CS. Data analysis: PY and SW. Statistical analysis: SW and PY. Manuscript preparation: PY and LC. Manuscript editing: PY and XZ. Manuscript review: NH. All authors contributed to the article and approved the submitted version.

## Funding

Roles of funding sources: Project (RDY2020-08) supported by Peking University People’s Hospital Scientific Research Development Funds.

## Conflict of Interest

The authors declare that the research was conducted in the absence of any commercial or financial relationships that could be construed as a potential conflict of interest.

## Publisher’s Note

All claims expressed in this article are solely those of the authors and do not necessarily represent those of their affiliated organizations, or those of the publisher, the editors and the reviewers. Any product that may be evaluated in this article, or claim that may be made by its manufacturer, is not guaranteed or endorsed by the publisher.

## Supplementary Material

Supplementary Figure 1 Bar chart shows the predictive values of RM5. Label 1 = metastatic tumor, Label 2 = chordoma, Label 3 = Ewing’s sarcoma, Label 4 = osteosarcoma, Label 5 = chondrosarcoma, Label 6 = neurogenic tumors, Label 7 = GCTs.

## Abbreviations

GCT, giant cell tumor; CT, computed tomography; RF, random forest; ICC, intra- and interclass correlation coefficients; RM, radiomics model; AUC, area under the receiver operating characteristic curve; ACC, accuracy.
